# Occurrence and Antibiogram of *Escherichia coli* O157 : H7 in Raw Beef and Hygienic Practices in Abattoir and Retailer Shops in Ambo Town, Ethiopia

**DOI:** 10.1155/2021/8846592

**Published:** 2021-04-01

**Authors:** Nega Desalegn Tadese, Endrias Zewdu Gebremedhi, Feleke Moges, Bizunesh Mideksa Borana, Lencho Megersa Marami, Edilu Jorga Sarba, Hirut Abebe, Kebede Abdisa Kelbesa, Dagmawit Atalel, Belay Tessema

**Affiliations:** ^1^College of Agriculture and Veterinary Science, Ambo University, Ambo, Ethiopia; ^2^College of Medicine and Health Sciences, University of Gondar, Gondar, Ethiopia

## Abstract

Foodborne infections are widespread and growing public health problems in the world. Shiga toxin-producing *Escherichia coli* O157 : H7 is one of the most significant foodborne pathogens. This study was conducted to assess the occurrence and antibiogram of *E. coli* O157 : H7 from raw beef as well as hygienic and sanitary practices of meat handling in abattoir and retailer shops. Systematic random sampling technique and census methods were used to collect samples from abattoir and retailer shops, respectively. All tryptone soya broth preenriched carcass samples were subcultured onto MacConkey agar. Then, the bacterium confirmed as *Escherichia coli* using biochemical tests was streaked onto Sorbitol-MacConkey agar and incubated at 37°C for 24 hrs. *Escherichia coli* O157 : H7 was confirmed by latex agglutination kit. In vitro antimicrobial susceptibility test of *Escherichia coli* O157 : H7 isolates was done against 13 antimicrobials. Hygiene and sanitation data were collected using a pretested structured questionnaire and observational checklist. Pearson Chi-square and Fisher's exact two-tailed tests were performed and differences were considered significant at *P* ≤ 0.05. Out of 197 meat samples, 23.4% (95% confidence interval (CI): 17.6–29.9%) and 9.1% (95% CI: 5.5–14.1%) were contaminated with *Escherichia coli* and *Escherichia coli* O157 : H7, respectively. There was a significant variation in the occurrence of *Escherichia coli* O157 : H7 between retailer shops (19.1%) and abattoir (7.2%) (*P* = 0.03). The study revealed that the municipal abattoir and retailer shops in Ambo town did not adhere to the required sanitation and hygienic standards. All *Escherichia coli* O157 : H7 isolates were susceptible to norfloxacin, sulfamethoxazole-trimethoprim, chloramphenicol, and ceftazidime. However, all isolates were resistant to amoxicillin. Multidrug resistance was widespread and was found in 66.3% of *Escherichia coli* O157 : H7 isolates. The occurrence of *Escherichia coli* O157 : H7 was high. Therefore, fulfilling national and international meat safety requirements, training and monitoring of meat handlers, and rational use of antimicrobials are recommended

## 1. Background

Foodborne diseases remain a challenging problem causing great human suffering and significant economic losses. While the burden of foodborne diseases is a public health concern globally, developing countries have the highest incidence and highest death rates [1]. The actual number of *Escherichia coli* (*E. coli*) O157 : H7 infections attributable to meat is difficult to assess accurately, because of the lack of diagnostic facilities and only a small proportion of illness cases are officially reported especially in developing countries [2]. However, a review of 16 articles and databases from 21 countries in Africa reported that the estimated global burden of *E. coli* O157 : H7 is 2,801,000 acute illnesses, 3890 cases of hemolytic uremic syndrome, and 230 deaths annually [3]. Foodborne diseases often follow the consumption of contaminated foodstuffs, especially from animal products such as meat from infected animals or carcasses contaminated with pathogenic bacteria [4]. *Escherichia coli* is among the most challenging *Enterobacteriaceae* group of bacterial meat contaminant worldwide. Most *E. coli* strains do not cause diseases and are actually part of the normal flora of the intestinal tract of animals and humans but detection of *E. coli* in foods intended for human consumption shows poor sanitary and hygiene during production, processing, transportation, or preparation [5]. However, there are a number of different pathogenic groups of *E. coli* that have been shown to cause various types of gastrointestinal infections, and deaths have been observed in humans. Among the enteric *E. coli*, shiga toxin-producing *E. coli* O157 : H7 is the most significant foodborne pathogens that have gained increased attention in recent years [6]. It has been the most commonly isolated serotype in association with abdominal cramps, bloody diarrhea, thrombotic thrombocytopenic purpura, hemorrhagic colitis, and hemolytic uremic syndrome in both outbreaks and sporadic cases [7].

Ruminants are regarded as the main reservoir of *E. coli* O157 : H7 though it has been isolated from other animal species such as pigs, gulls, geese, and pet animals [8]. Food is the predominant transmission route of *E. coli* O157 : H7 which is responsible for more than 52% of outbreak-related cases in the United States. Beef is the most common vehicle among foodborne outbreaks of *E. coli* O157 : H7 [9]. It can be contaminated through contact with the animal's skin and hair, limbs, blood, stomach, gut contents, bile and, equipment, hands, and worker's clothes [10]. Bacterial contamination of the feces/hide can be transferred onto previously sterile meat surfaces during slaughtering and dressing especially when slaughtering is performed on the floor with the absence of a carcass suspension system and careless evisceration that spreads intestinal content onto the meat surface [11].

Resistance to antimicrobial is highly prevalent in bacterial isolates worldwide, particularly in developing countries [12]. Antimicrobials are used in food animals to prevent, control, and treat disease and to promote the growth of food-producing animals. The increased use of antimicrobial agents in food animal production and human is a significant factor in the emergence of antimicrobial-resistant bacteria [13]. A review of 40 years of enteric antimicrobial resistance research in Eastern Africa states that *E. coli* O157 : H7 is potential for zoonotic transmission to humans and has developed high rates of resistance to available treatment regimens [14]. Meat is a major source of transmission of antimicrobial-resistant organisms to humans causing disease [15]. Furthermore, this situation is complicated by the potential of resistant bacteria to transfer their resistance determinants to resident constituents of the human microflora and other pathogenic bacteria [15, 16].

In developing countries, there is a food safety knowledge gap and animals are commonly slaughtered and dressed under unhygienic conditions [17, 18]. Ethiopia is one of the developing sub-Saharan African countries sharing the high burden of diarrheal morbidity and mortality [19]. Information about human infections with *E. coli* O157 : H7 is limited in this country; nevertheless, in a study conducted on 422 diarrheic children under 5 years in the northern part of Ethiopia, 59 (28.9%) of the children were positive for *E. coli* O157 : H7 [20]. The habit of consuming raw and/or undercooked meat is one of the factors that exacerbate the transmission of foodborne pathogens including *E. coli* O157 : H7 in the country. Sufficient heating of meat kills these organisms [21]. However, consumption of raw or undercooked beef in the form of “*kitfo*” (minced raw beef mixed with a chili powder-based spice blend and a clarified butter infused with herbs and spices), “*leb*-*leb*” (undercooked “*kitfo*”), “*gored*-*gored*” (cuts of raw meat with butter and pepper), and “*kurt*” (raw beef consumed with hot pepper and mustard) is common cultural practices in Ethiopia [22].

In Ethiopia, the few studies conducted on *E. coli* O157 : H7 showed prevalence ranging from 2.3% to 10.4% [22–25]. However, studies on the hygiene and sanitation practices in meat processing establishments are lacking. Therefore, the aim of the present study was to investigate the prevalence of *E. coli,* occurrence and antibiogram of *E. coli* O157 : H7 from raw beef, and hygienic and sanitary practices of meat handling in abattoir and retailer shops in Ambo town, West Shewa Zone, Ethiopia.

## 2. Methods

### 2.1. Study Design and Study Area

A cross-sectional study design was employed for the purpose of this study. There are 31 legally registered retailer shops and 1 municipal abattoir in Ambo town during the study period. All retailer shops receive carcass from Ambo municipal abattoir. Ambo town is the administrative center of West Shewa Zone. It is located at latitude and longitude of 8°59′N 37°51′E8.983°N 37.85°E, respectively, and at an elevation of 2101 meters above sea level and 114 Km West of Addis Ababa, the capital of Ethiopia.

### 2.2. Sample Size Determination

Sample size for this study was determined using single population proportion standard formula.

(1) $$\begin{array}{c} n=\frac{Z_{\alpha /2}2\;P\left(1-P\right)}{d^{2}}, \end{array}$$

*Z* is *z* statistic for level of confidence, *n* is the required sample size, *P* is the expected prevalence, and *d* is desired absolute precision.

Previous study done in abattoir and retailer shops in Addis Ababa showed the prevalence of *E. coli* O157 : H7 to be 13.3% [22]. Therefore, using 13.3% expected prevalence, at a confidence level of 95% and required absolute precision of 5%, the minimum calculated sample size was 174. But 197 samples were taken deliberately in order to maximize the precision of the study. Out of the total samples collected, 166 and 31 meat samples were from Ambo municipal abattoir and retailer shops, respectively.

### 2.3. Questionnaire Survey

A pretested structured questionnaire and observational checklists were used to collect the necessary field-level data. They were designed after reviewing relevant literature, national and international guidelines to obtain hygienic status, and practices in abattoir and retailer shops. Structured questionnaire interview was used to collect data from 31 retailers (one from each retailer shop) and all 14 abattoir workers who are directly involved in slaughtering, evisceration, and carcass splitting. The questionnaire was developed to gather data about sociodemographic characteristics, meat handling experience, training on meat safety, status of medical screening and certiﬁcation, knowledge about foodborne disease, and hygienic practices of workers regarding meat safety in the abattoir and retailer shops. Observational checklist was used to collect data regarding housing (floor, roof, and ceiling) of retailer shops and abattoir, availability of cooling materials, tap water, hot water, retention room, and bathroom in meat handling places. Additionally, practices like slaughtering, evisceration, splitting, loading, and transportation of carcass in the abattoir were included.

### 2.4. Sample Collection Procedure

First, animals were selected using a systematic random sampling technique from a list of animals that were brought to Ambo municipal abattoir. Then, raw cut of meat samples was collected from specific sites (neck, brisket, fore rib, flank, and rump) of a carcass [26]. Similarly, raw meat samples from the same sites of carcasses were collected from all meat retailer shops in Ambo town (*n* = 31). All samples from different retailer shops and abattoir were placed in separate sterile plastic bags (Seward, England), labeled with identification number, and immediately transported to the Ambo University Zoonoses and Food Safety Laboratory in an icebox with ice packs and processed within 4 hrs.

### 2.5. Sample Preparation and Isolation Procedure

Raw meat samples collected from abattoir and retailer shops were taken out of plastic bags using sterile thumb forceps. From each chopped and mixed meat sample, 25 gm was transferred into a sterile stomacher bag (Seward, England), containing 225 ml of tryptone soya broth (TSB) (Himedia, India) and homogenized using homogenizer (Stomacher 400, Seward Medical, England) at 260 RPM for 2 minutes. The resulting homogenate was incubated at 37°C for 24 hrs. All preenriched meat samples were subsequently subcultured onto MacConkey agar (Himedia, India) and incubated at 37°C for 24 hrs. Five to ten suspected colonies of *E. coli* (pinkish color appearance) were subcultured onto separate nutrient agar (Himedia, India) and confirmed by biochemical tests: fermentation of lactose and glucose using triple sugar iron agar, hydrogen sulfide (H_2_S) negative, production of indole (positive), methyl red test (positive), Voges-Proskauer test (negative), and Simon citrate agar test (negative) were considered as *E. coli*. Then the bacterium confirmed as *E. coli* was streaked onto Sorbitol-MacConkey agar (Himedia, India) and incubated at 37°C for 24 hrs. Nonsorbitol fermenting (colorless) isolates were passed for serological typing.

### 2.6. Serological Test

All nonsorbitol fermenting colonies from the Sorbitol-MacConkey agar were serologically confirmed using *E. coli* O157 : H7 latex agglutinations assay (Abraxis LLC, USA), containing latex particles coated with antibodies specific for *E. coli* O157 : H7 antigen. Identification of *E. coli* O157 : H7 was carried out following the manufacturer's instruction. Nonsorbitol fermenting isolates were inoculated onto nutrient agar for serological testing. Using one of the provided transfer pipettes, one drop of peptone buffered saline (PBS) was placed onto one (1) circle on the test card. A portion of a suspected colony from the agar plate was picked using single used sterile plastic sticks and emulsified thoroughly in the drop of PBS in one of the circles. One free falling drop (with vial held vertically) of the *E. coli* O157 : H7 Latex Antibody bead reagent was dispensed onto each circle and the test card rotated using a complete circular motion for up to one minute or until agglutination was evident; whichever occurs first, the results were recorded. Agglutination of the test latex within one minute was considered as a positive result. This indicates the presence of *E. coli* serogroup O157 : H7. The absence of agglutination occurring within one minute was considered a negative result. This indicates the absence of *E. coli* serogroup O157 : H7.

### 2.7. Antimicrobial Susceptibility Test

*E. coli* O157 : H7 isolates were subjected to in vitro susceptibility test against 13 commonly used antimicrobial drugs using the disk diffusion method following guidelines established by the Clinical and Laboratory Standards Institute (CLSI) [27]. Test suspension was prepared from a pure culture of *E. coli* O157 : H7 isolates, inoculated into a test tube of 5 ml TSB (Himedia, India), and incubated at 37°C for 6 hrs. The bacterial suspension was adjusted to 0.5McFarland turbidity standards. Mueller-Hinton agar (Bacton Dickinson, USA) plates were prepared according to the guidelines of the manufacturer. The diluted bacterial suspensions were swabbed in three directions uniformly on the surface of Mueller-Hinton agar plates using sterile cotton swabs. After the plates dried, with the aid of sterile thumb forceps, antibiotic-impregnated disks (Oxoid, England) were placed to the surface of the inoculated plates. Then, the plates were incubated aerobically at 37°C for 24 hrs. Finally, the diameter of the inhibition zone formed around each disk was measured on black surface using a transparent ruler by placing it over the plates. The results were classified as sensitive, intermediate, and resistant according to the CLSI [27].

### 2.8. Quality Control

Confidences in the reliability of test results were increased by adequate quality assurance procedures and the routine use of control strains. Thus, *E. coli* ATCC-25922 (susceptible to all tested drugs) was taken as an important part of quality control for culture and antimicrobial susceptibility tests. The sterility of sample collecting materials was checked randomly by culturing on nutrient agar and sterility of culture media was checked by incubating from each batch of prepared media for 24 hrs. Moreover, the whole procedures and result interpretation were done following standard operating procedure (SOP). The questionnaire was daily checked by the principal investigator for its completeness.

### 2.9. Data Management and Statistical Analysis

Questionnaire and laboratory data were entered into a Microsoft Excel spreadsheet. SPSS 20 statistical software (SPSS Inc., Chicago, IL, USA) was used for analyses of data. Descriptive statistics such as frequencies were used to present the findings of the questionnaires. The percent occurrence of *E. coli* O157 : H7 in beef samples was estimated using a formula, that is, the number of positive samples divided by the total number of samples examined multiplied by 100. The binomial exact method was used to calculate the 95% confidence interval (CI) of the prevalence estimates. *P*-value ≤ 0.05 was considered statistically significant.

## 3. Results

### 3.1. Prevalence of *E. coli* and Occurrence of *E. coli* O157 : H7

Out of 197 samples tested, 23.4% (95% CI: 17.6–29.9%) and 9.1% (95% CI: 5.5–14.1%) were found to be contaminated with *E. coli* and *E. coli* O157 : H7, respectively. Twelve samples from abattoir (7.2%) and 6 samples from retail shops (19.4%) had *E. coli* O157 : H7 (Table 1).

### 3.2. Sociodemographic Characteristics of Retailer Shops and Abattoir and Workers

The results of the sociodemographic information of abattoir and meat retailer men interviewed in the abattoir and retailer shops in Ambo are shown in Table 2. The results revealed that all personnel working in the establishments are male. Most of the respondents from abattoir (42.9%) and from retailer shops (41.9%) were between the ages of 21 and 30 years.

### 3.3. Professional Experience, Training, Health Evaluation, and Awareness about Foodborne Disease of Study Participants

Information regarding medical test, training, and professional experience of the interviewed workers is shown in Table 3. The majority (64.5%) of respondents did not know about their health status whether they are healthy enough to work in meat processing. More than half of the abattoir workers (54.8%) and meat retailer men (57.1%) have been working in the establishments for less than six years. The majority of retailer shops (83.5%) and abattoir workers (71.4%) did not receive formal training for sanitary and hygienic handling of meat.

### 3.4. Personal Hygiene and Sanitation of Workers regarding Meat Safety

All the respondents from retailer shops indicated that they always clean their hands before meat handling and use reusable cloth towel to clean equipment and dry hands. About 35.5% of the respondents indicated that they do not wear a gown, but instead wear casual (street) clothes. About 51.6% of the respondents indicated that they do not remove their jewelry during meat handling. Most of the study participants (83.9%) wash their hands using cold water and soap. Similarly, 83.9% of respondents wash equipment (knife, ax, balance, etc.) every day at the end of the process. Seventy-four percent of the meat retailers wash meat using cold water when there is visible contamination, whereas 25.8% of them do not wash even if there is contamination, either they cut and remove or leave it as it is (Table 4).

### 3.5. Facilities and Hygienic Practices in Ambo Municipal Abattoir

**Figure 1:**
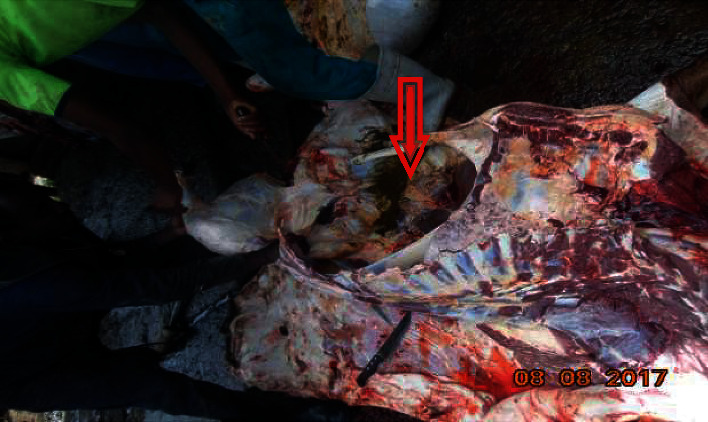
Contamination of carcass by visceral content at Ambo municipal abattoir.

The surroundings of abattoir house were full of leftover dirty materials and doors were always open without any restriction on personal movement to go inside and out of slaughterhouses. The floor of abattoir was made of concrete and impervious but has no ceiling. There was no hot water, adequate supply of tap water, sterilizer, retention room (cooling facilities), change rooms, and bathroom facilities in the abattoir. Evisceration and carcass splitting takes place on the floor often not clean (Figure 1). Animals were not washed before slaughtering and there was no separation between dirty and clean areas in the abattoir. Workers were not interested in washing their hands, knife, and axes during slaughtering process and they were not interested in preventing leakage from the anus or bursting of the visceral contents to sterile carcass. There is one meat inspector, but slaughtering happens in the absence of the meat inspector.

### 3.6. Antimicrobial Susceptibility of *E. coli* O157 : H7

**Figure 2:**
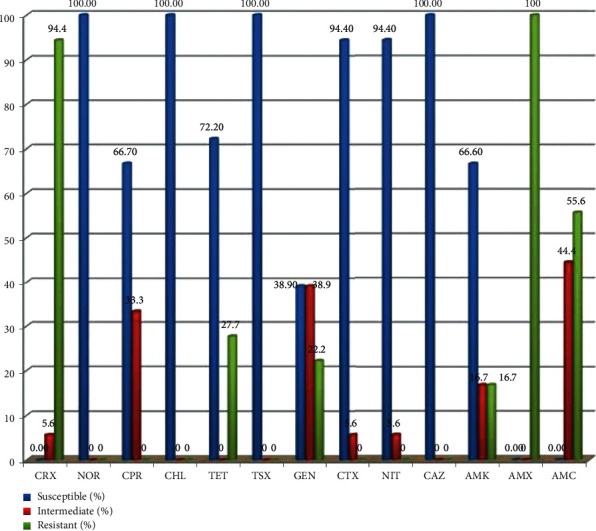
Antimicrobial susceptibility test result of *E. coli* O157 : H7 isolated from raw beef at abattoir and retailer shops in Ambo town, West Shewa, Ethiopia. CRX: cefuroxime, NOR: norfloxacin, CPR: ciprofloxacin, CHL: chloramphenicol, TET: tetracycline, TSX: trimethoprim-sulfamethoxazole, GEN: gentamycin, CTX: cefotaxime, NIT: nitrofurantoin, CAZ: ceftazidime, AMK: amikacin, AMX: amoxicillin, and AMC: amoxicillin-clavulanate.

All *E. coli* O157 : H7 isolates were subjected to antimicrobial susceptibility test using 13 selected antimicrobial drugs. The isolated strains were pan susceptible (100% susceptible) to norfloxacin, trimethoprim-sulfamethoxazole, chloramphenicol, and ceftazidime. Additionally, 72% and 66.7% of the isolates were susceptible for tetracycline and ciprofloxacin respectively. All *E. coli* O157 : H7 isolates were resistant to amoxicillin (100%) followed by cefuroxime (94.4%), amoxicillin-clavulanate (55.6%), tetracycline (27.7%), and gentamicin (22.2%) (Figure 2).

From total of 18 E*. coli* O157 : H7 isolates, 12 (66.3%) were found to be resistant to three or more antimicrobial drugs, that is, multiple drug resistance (MDR). The most frequently observed resistance combinations were cefuroxime and amoxicillin (33.3%) (Table 5).

## 4. Discussion

The present study was conducted to assess the occurrence of *E. coli* O157 : H7 and its antimicrobial susceptibility on meat samples collected from an abattoir and retailer shops in Ambo town. The carcass contamination with *E. coli* in the retailer shops and abattoir was 23.4%. This is in agreement with the result of 24.8% reported in Dire Dawa [28] and 27.3% in Mekele municipality abattoir in northern Ethiopia [17]. The prevalence of *E. coli* in the present study was lower than the 46.5% reported in Nigeria [29].

Even though the present finding is lower than the report from Nigeria, it confirmed the high rate of contamination of meat with *E. coli* due to unhygienic practices, which is also an indication of the presence of unacceptable levels of other pathogenic microorganisms.

The occurrence of *E. coli* O157 : H7 (9.1%) from bovine meat at both the abattoir and raw meat retailer shops was in line with the previous study from Tigray 10.4% [30], Debre Zeyit 8% [23], and Addis Ababa 10.2% [22]. Additionally, comparable results were also reported in other parts of the world such as 8.9% [31] and 9.6% [32] in Iran, 8.8% [33] in South Africa, and 13.3% [34] in China.

However, the present finding is higher than some other reports from Ethiopia, such as 1.3% in Addis Ababa [25], 2.7% in Haramaya University [24], and 6.7% in Mekele [35], and also from other countries in the world, such as 3.76% in Botswana [36], 1% in Ireland, 0.3% in the Czech Republic, and 0.3% in the Netherlands [37]. On the other hand, other scholars reported much higher results than the present findings: 60% from street meat sellers in Mekele town and 19.8% and 53% in Nigeria [38]. The overall variations in the prevalence of *E. coli* O157 : H7 might be due to the difference in sample size, sampling techniques, laboratory methodologies, study areas, time, and hygienic conditions employed [26, 39].

With regard to meat, the source of the significantly higher contamination of *E. coli* O157 : H7 was found at the retailer shops (19.1%) than the abattoir (7.2%). The higher occurrence of *E. coli* O157 : H7 in retailer shops could be due to the risk of carcass cross-contamination during transportation in a car and handling of meat at retailer shops. Ambo municipal abattoir has only one vehicle used to transport meat from the abattoir to retailer shops. Though respondents mentioned that they clean the vehicle daily, it was not clean as per our observation. Abattoir workers carry the meat on their back or hold it using their two hands supporting through their chest. Therefore, a higher prevalence of *E. coli* O157 : H7 at the retailer shops could be as a result of unhygienic handling practice of meat at retailer shops and direct contact between contaminated clothes of workers and carcass. Furthermore, there is a great possibility of bacterial cross-contamination due to direct contact of different carcasses while transporting together in a single vehicle.

The current study noted that 64.5% of workers in both abattoir and retail shops had no medical test and health certificate. Similar reports from Mekele [17] and Egypt [40] also noted that upon inspection most workers did not have valid health certificates. Shortcomings observed in the implementation of personal hygiene practices can be addressed by proper training, education, and monitoring of the workers [41]. In addition to this, regular updating and refresher courses should be carried out more frequently. This will help the meat handlers to have a better understanding of risks associated with contamination of meat with potential pathogens and sanitation practices [42]. Most respondents (83.9%) in the present study did not receive any formal training regarding hygiene and sanitation of meat handling neither prior nor after employment. This critical violation is comparable to the proportions of respondents of other studies who also indicated that they did not receive training [41, 43]. Reports from Pretoria, South Africa [43], Western Romania [41], and Alexandria, Egypt [40], have shown that most meat handlers lack meat safety knowledge and adequate training and are frequently engaged in poor handling practices. Studies also highlighted that individuals with proper professional training regarding meat safety significantly do better practices compared to the untrained [41, 42]. This shows that the quality of practices is improved mainly by professional training.

Meat contamination during processing is partly due to a lack of knowledge as to how to improve conditions in meat industry [44]. About 45.5% of the retailers and 64% of the abattoir workers knew that contaminated meat can cause bacterial diseases. This study showed that 32.3% of retail workers and 57.1% of abattoir workers were able to name foodborne bacterial diseases and some of their signs and symptoms. Workers are at risk of meat contamination and should self-report when ill [44]. In this study, a large proportion of meat handlers from retailers (38.7%) and abattoir (42.9%) did not report any illness to the supervisors. Another previous study showed that 96.4% of the respondents report their illness to supervisors and visit the nearby health facility timely to get appropriate diagnosis and treatment [43], whereas in the present study 61.3% of the retailer and 57.1% of abattoir workers indicated that they go to clinic or hospital when sick. Reporting and taking a leave when sick is very important when working on the food premises to prevent chances of contamination [44]. However, in our study, as most of the participants were temporary workers paid on a daily basis, they were unlikely to report illness and take time off. These conditions can lead to pathogen contamination of meat from meat handlers.

Contamination of meat and meat handlers could be prevented by wearing protective clothes [45]. About 35.5% of the respondents indicated that they did not wear protective clothing. Out of those who wear protective clothes, 30% of respondents' clothes were not clean. Haileselassie et al. [17] also recorded that a larger proportion of workers from selected butcher shops in Ethiopia operate without wearing protective clothing. In Kenya, less than 50% of workers wore protective clothing at all times [46]. In contrast to our study, Nel et al. [43] reported that all respondents declared that they always wear protective clothes. Working clothes should be cleaned every day [47]. However, in this study, only 27.5%, of respondents indicated that they wash their protective clothing daily. Therefore, the workers need to be properly trained and provided with adequate protective clothing in order to prevent possible chances of cross-contamination.

In this study, all respondents indicated that they always clean their hands before meat handling. In addition, upon asking the workers what they use for handwashing, 83.9% indicated that they used soap and cold water. Damp hands can result in skin excoriation leading to a higher number of types of bacterial colonization and facilitate the spread of pathogens [48]. Our observation during the study showed that the retailer operators retailing meat also wiped their hands, cutting board, and scale surfaces with a dirty reusable cloth. The piece of cloth used was not frequently washed or changed during the day. Even though the intention was good, the wiping cloth was reused the whole day and can accumulate microorganisms that can be transferred to the retailer operators' hands, to utensil surfaces, and finally to meat. Soft, absorbent paper towels are recommended for drying hands than the use of a cloth. Clothes have been reported to be ineffective in removing microorganisms, thereby increasing the chance of cross-contamination [49].

In the current study, the majority (93.5%) of retailer shops' floor was constructed of concrete. All of the walls of the shops were painted with white and red color. Nevertheless, 35.5% of the retailer shops floor had cracks, and 19.4% of the shops do not have a ceiling which further hinders cleaning. These conditions in the present study settings disagree with the WHO and FAO standards [50].

The surrounding of the slaughterhouse is full of leftover dirty materials (gastrointestinal content, horn, shank, and bones) collected from daily slaughtered animals. Moreover, gates of abattoir are always opened without any restriction on personal movement to go inside and out of slaughterhouses. This may create a favorable condition for transferring *E. coli* O157 : H7 from the environment to slaughterhouse. The floor of the abattoir should be hard concrete and impervious, to reduce dirt in the slaughterhouse and allow drainage and ease of cleaning. Similarly, a roof is important to protect the carcass from the weather and to reduce the temperature in the slaughterhouse [50]. In the present study, floor of the abattoir is made of concrete and impervious but has no ceiling. Even though washing of the abattoir floor takes place every day at the end of slaughtering process, the wall is not cleaned and washed at the end of the working day.

According to international guidelines, hot and cold water, sanitizer, and retention room should be readily accessible for cleaning equipment and workers' hands [51]. There were no hot water, adequate supply of tap water, sterilizer, retention room (cooling facilities) change rooms, and bathroom facilities in Ambo abattoir. From the observations made in the current study, there is no separation between dirty and clean areas. Both slaughter and postmortem operation are conducted in the same place and bleeding evisceration and carcass splitting take place on the ground in the same area, which leads to high possibilities of contamination during dressing of carcass from the skin, the intestines, and the ground. This is contrary to the recommendations of the Codex Alimentarius Commission [51] and Norrung and Buncic [52]. Any visual contaminations on the carcasses were removed by washing.

Unlike the reports of Gill et al. [53], Nastasijevic et al. [54], and Blagojevic et al. [55], this study confirmed that there was no washing of animals before slaughtering, “bagging” of anus and tying “rodding” of esophagus before evisceration. Feces adhering to the animals can be carried into the abattoir on the hair, hide, hooves, and tail of the animal and can become a major source of carcass contamination. Additionally, workers in the abattoir had no intention for personal hygiene and were not interested in washing their hands, knife, and axes during the slaughtering process. They were not concerned to prevent leakage from the anus or bursting of the visceral contents to sterile carcass. Their hands and clothes were not clean throughout the working days.

Even though there is one veterinary meat inspector in the abattoir, it was observed that many of the activities in the abattoir happen in the absence of the meat inspector. This is in violation of the stipulations; a licensed inspector must perform antemortem and postmortem inspection and must be present when slaughtering is being conducted for meat intended for commercial purposes [56].

Antimicrobial resistance has been recognized as an emerging worldwide problem in human and veterinary medicine in both developed and developing countries. The increased use of antimicrobial agents in food animal production and human is a significant factor in the emergence of antibiotic-resistant bacteria [13]. Antimicrobial resistance of *E. coli* O157 : H7 isolates from animal and human sources have been reported from central Ethiopia [23]. In the present study, all of the 18 isolates were susceptible to norfloxacin, trimethoprim-sulfamethoxazole, chloramphenicol, and ceftazidime. This is consistent with the findings of Rahimi and Nayebpour in Iran [57] and Bekele et al. [22] in Ethiopia. On the other side, the current study revealed that isolates were resistant to amoxicillin (100%), cefuroxime (94.4%), ciprofloxacin (66.7%), amoxicillin-clavulanate (55.5%), tetracycline (27.7%), and gentamicin (22.2%). A study carried out in Saudi Arabia revealed a similar finding that there was a resistant strain to the drugs such as tetracycline and ciprofloxacin. A similar percentage of resistance to amoxicillin and tetracycline has been reported previously in Ethiopia [24]. The significantly high level of antimicrobial resistance was probably an indication of their extensive usage either in the public health sector or in the food-producing animals or both for the therapeutic purpose of *E. coli* and other infections.

Multiple antimicrobial resistance may be acquired through mobile genetic elements such as plasmids, transposons, and class 1 integrons [58]. The present study showed that 66.3% of the *E. coli* O157 : H7 isolates were resistant to three or more classes of antimicrobials. This finding was lower than the previous findings in Addis Ababa [25] and in Mekele [30]. Additionally, it was lower than with the findings of other researchers, who reported MDR among *E. coli* O157 : H7 isolates [59–61]. The occurrence of MDR in this study was higher than the findings of other studies conducted in Addis Ababa (22.6%) [23]. This finding is comparable to the previous finding in Haramaya (66.7%) [24]. The occurrence of MDR observed in this study might be due to the administration of multiple antimicrobials for prophylaxis or infection control and indiscriminate use of antimicrobials in the farms and/or public health sector, thereby selecting for resistant populations of *E. coli* O157 : H7.

The occurrence and multidrug resistance of *E. coli* O157 : H7 in this study imply an unacceptable level of hygiene and sanitation practice in meat handling and irrational use of antimicrobials. The high contamination of meat by MDR *E. coli* O157 : H7 and widespread habit of raw beef consumption in Ethiopia [22] call a concern for a potential outbreak of drug-resistant human pathogens for customers who regularly consume raw meat in the study area.

## 5. Limitation of the Study

Quantitative analysis of microbial load of meat in the abattoir and retailer shops was not performed. Isolation of *E. coli* O157 : H7 was done without selective enrichment and selective media. The result in this study indicates a relatively high frequency of *E. coli* 157:H7. However, the percentage of *E. coli* 157:H7 reported in this study might be an underestimate due to the chances of losing the specific pathogen from a diversity of lactose-fermenting bacteria.

## 6. Conclusions

The present study revealed a relatively high occurrence of *E. coli* O157H7. *Escherichia coli* O157 : H7 isolates developed drug resistance to most antimicrobials tested. All of the *E. coli* O157 : H7 isolates showed MDR. The study revealed that municipal abattoir and retailer shops in Ambo town did not adhere to the required sanitation and hygiene standards. Proper training and monitoring of meat handlers will help to ensure sanitation and hygienic meat handling practices to provide good quality wholesome meat. The municipal abattoir and retailer shops in the study area should adhere to national and international guidelines. There is a need to emphasize the rational use of antimicrobials in agriculture and medicine. In addition, regular antimicrobial susceptibility surveillance is essential. Further research is recommended to validate the source and point of contamination.

## Figures and Tables

**Table 1:** Prevalence of *E. coli* and occurrence of *E. coli* O157 : H7 in beef from abattoir and retail shops in Ambo, West Shewa, Ethiopia (January 2016 to May 2017).

Prevalence of *E. coli* and occurrence *E. coli* O157 : H7					
Sources of meat	№ examined	*E. coli* positive (%)	*P*-value	*E.coli* O157 : H7 positive (%)	*P*-value
Abattoir	166	32 (19.3)	0.002	12 (7.2)	0.031
Retail shops	31	14 (45.2)		6 (19.4)	

Total	197	46 (23.4)		18 (9.1)	

*E. coli* *=* *Escherichia coli,* № = number, % = percent, and *P* = probability.

**Table 2:** Sociodemographic characteristics of retailer shops and abattoir workers in Ambo, West Shewa, Ethiopia (January 2016 to May 2017).

Variables	Categories	Retailer shop workers (*n* = 31)		Abattoir workers (*n* = 14)	
		Frequency	Percent (%)	Frequency	Percent (%)
Age in years	< 21	2	6.5	3	21.4
	21–30	13	41.9	6	42.9
	31–40	7	22.6	4	28.6
	> 40	9	29.0	1	7.1
Religion	Orthodox	25	80.6	11	78.6
	Protestant	6	19.4	3	21.4
Educational status	Illiterate	3	9.7	2	14.3
	Primary	18	58.1	7	50.0
	Secondary	9	29.0	3	21.4
	Tertiary	1	3.2	2	14.3
Employment status	Temporary	25	80.6	9	64.3
	Permanent	6	19.4	5	35.7

**Table 3:** Professional experience, training, health evaluation, and awareness about foodborne disease of retailer shop and abattoir workers in Ambo, West Shewa, Ethiopia (January 2016 to May 2017).

Variables	Categories	Retailer men		Abattoir workers	
		Frequency	%	Frequency	%
Medical test	Yes	11	35.5	5	35.5
	No	20	64.5	9	64.5
Health certificate	Yes	11	35.5	4	28.6
	No	20	64.5	10	71.4
Training	Yes	5	16.5	4	28.6
	No	26	83.5	10	71.4
Work experience	0–5	17	54.8	8	57.1
	6–10	8	25.8	3	21.4
	11–15	6	19.4	3	21.4
Disease causing bacteria can be found in contaminated meat	Yes	14	45.2	9	64.0
	No	17	54.8	5	35.7
Knowledge of food born bacteria	Yes	10	32.3	8	57.1
	No	21	67.7	6	42.9
Knowledge of sign and symptoms of enteric bacterial diseases	Yes	9	29.0	8	57.1
	No	22	71.0	6	42.9
Action when sick	Go to work	19	61.3	8	57.1
	Report to head	12	38.7	6	42.9
Seeking medication	Self-medication	4	12.9	4	28.6
	Go to pharmacy	5	16.1	2	14.3
	Go to clinic or hospital	22	71.0	8	57.1

**Table 4:** Personal hygiene and sanitation of retailers regarding meat safety in Ambo, West Shewa, Ethiopia (January 2016 to May 2017).

Variables	Meat retailer men		
	Categories	Frequency	%
Wear gown	Yes	20	64.5
	No	11	35.5
Removal of jewelry	Yes	15	48.4
	No	16	51.6
Time of hand washing	Before, between, and after meat handling	11	35.5
	Before and after meat handling	20	64.5
Hand washing using	Cold water only	5	16.1
	Soap and cold water	26	83.9
Frequency of washing equipment	When it became dirty	3	9.7
	Every day at the end of process	26	83.9
	Two times per week	2	6.5
Frequency of washing surface	Every day at the end of the process	21	67.7
	Two times per week	4	12.9
	Once per week	6	19.4
Frequency of washing protective cloths	Daily	8	25.8
	Once a week	11	35.5
	Two times a week	9	29.0
	Three times a week	3	9.7
Carcass washing	Yes	23	74.2
	No	8	25.8
Refrigerator available	Yes	13	42.0
	No	18	58.0
Floor constructed of	Concrete	29	93.5
	Earthen materials	2	6.5
Floor free from crack	Yes	20	64.5
	No	11	35.5
Having ceiling	Yes	25	80.6
	No	6	19.4

**Table 5:** Multidrug resistance pattern of *E. coli* O157 : H7 from abattoir and retailer shops in Ambo, West Shewa, Ethiopia (January 2016 to May 2017).

Multidrug resistance pattern of *E. coli* O157 : H7			
Antibiotics	No. of combinations	Frequency	Percent (%)
CRX and AMX	2	6	33.3
CRX, AMX, and AMC	3 (MDR)	4	22.2
CRX, AMX, and TET	3 (MDR)	1	5.5
GEN, AMX, and AMC	3 (MDR)	1	5.5
CRX, AMX,TET, and AMC	4 (MDR)	1	5.5
CRX, AMX, AMC, and AMK	4 (MDR)	1	5.5
CRX, AMX, TET, and AMC	4 (MDR)	1	5.5
CRX, AMK, TET, GEN, and AMC	5 (MDR)	2	11.1
CRX, AMX, AMK, GEN, and AMC	5 (MDR)	1	5.5
Total		18	100

AMK: amikacin, AMX: amoxicillin, AMC: amoxicillin-clavulanate, CRX: cefuroxime, GEN: gentamycin, MDR: multidrug resistance, and TET: tetracycline.

## Data Availability

All relevant data are included within the paper. The dataset is available from the corresponding author and can be obtained upon reasonable request.

## Abbreviations

ATCC: American Type Culture
CLSI: Clinical and Laboratory Standards Institute
CI: Confidence interval
*E. coli*: *Escherichia coli*
MDR: Multidrug resistance
SOP: Standard operating procedure
TSB: Tryptone soya broth.

## References

[1] Havelaar A. H., Kirk M. D., Torgerson P. R. (2015). World health organization global estimates and regional comparisons of the burden of foodborne disease in 2010. *PLoS Medicine*.

[2] Scallan E., Hoekstra R. M., Angulo F. J. (2011). Foodborne illness acquired in the United States-major pathogens. *Emerging Infectious Diseases*.

[3] Shannon E. M., Elaine S., Andria J. B. (2014). Global incidence of human Shiga toxin–producing *Escherichia coli* infections and deaths: a systematic review and knowledge synthesis. *Foodborne Pathogens and Disease*.

[4] Loretz M., Stephan R., Zweifel C. (2011). Antibacterial activity of decontamination treatments for cattle hides and beef carcasses. *Food Control*.

[5] Park S., Ryu S., Kang D. (2011). Improved selective and differential medium for isolation of *Escherichia coli* O157: H7. *Journal of Clinical Microbiology*.

[6] Pal M. (2007). *Zoonoses*.

[7] Karmali M. A., Gannon V., Sargeant J. M. (2010). Verocytotoxin-producing *Escherichia coli* (VTEC). *Veterinary Microbiology*.

[8] Gyles C. L. (2007). Shiga toxin–producing *Escherichia coli*. *Journal of Animal Science*.

[9] Rangel J. M., Sparling P. H., Crowe C., Griffin P. M., Swerdlow D. L. (2005). Epidemiology ofEscherichia coliO157:H7 outbreaks, United States, 1982-2002. *Emerging Infectious Diseases*.

[10] Eugène N., Martin P. O., Anastase K., Marianne S. (2015). Risk factors and control measures for bacterial contamination in the bovine meat chain: a review on *Salmonella* and pathogenic *E.coli*. *Journal of Food Research*.

[11] Sofos J. N. (2008). Challenges to meat safety in the 21st century. *Meat Science*.

[12] Aibinu I. E., Peters R. F., Amisu K. O., Adesida S. A. (2007). Multidrug resistance *in E. coli* O157 strains and public health implication. *Journal of Animal Science*.

[13] Antibiotic Resistance Threats in the United States of America in 2013 https://www.cdc.gov/drugresistance/threat-report-2013/pdf/ar-threats-2013-508.pdf#page=36 25162160

[14] Omulo S., Thumbi S. M., Njenga M. K., Call D. R. (2015). A review of 40 years of enteric antimicrobial resistance research in Eastern Africa: what can be done better?. *Antimicrobial Resistance and Infection Control*.

[15] Johnson J. R., Murray A. C., Gajewski A. (2003). Isolation and molecular characterization of nalidixic acid-resistant extraintestinal pathogenic *Escherichia coli* from retail chicken products. *Antimicrobial Agents and Chemotherapy*.

[16] Österblad M., Hadanen A., Manninen R., Leistevuo T., Peltonen R., Meurman O. (2004). A between-species comparison of antimicrobial resistance in enterobacteria in fecal flora. *Journal of Antmicrob Chemotherapy*.

[17] Haileselassie M., Taddele H., Adhana K., Kalayou S. (2013). Food safety knowledge and practices of abattoir and butchery shops and the microbial profile of meat in Mekelle City, Ethiopia. *Asian Pacific Journal of Tropical Biomedicine*.

[18] Bello M., Lawan M. K., Aluwong T., Sanusi M. (2015). Management of slaughter houses in northern Nigeria and the safety of meat produced for human consumption. *Food Control*.

[19] Global Burden of Diseases, Injuries, and Risk Factors Study 2010. Ethiopia GDB Profile. healthmetricsandevaluation.org. [http://www.healthdata.org/sites/default/files/files/country_profiles/GBD/ihme_gbd_country_report_ethiopia.pdf]

[20] Adugna A., Kibret M., Abera B., Nibret E., Adal M., Antibiogram of E. (2015). Antibiogram of *E. coli* serotypes isolated from children aged under five with acute diarrhea in Bahir Dar town. *African Health Sciences*.

[21] Doyle M. P. (1991). *Escherichia coli* O157: H7 and its significance in foods. *International Journal of Food Microbiology*.

[22] Bekele T., Zewde G., Tefera G., Feleke A., Kaleab Z. (2014). *Escherichia coli* O157:H7 in raw meat in Addis Ababa, Ethiopia: prevalence at an abattoir and retailers and antimicrobial susceptibility. *International Journal of Food Contamination*.

[23] Hiko A., Asrat D., Zewde G. (2008). Occurrence of *Escherichia coli* O157:H7 in retail raw meat products in Ethiopia. *Journal of Infectious Disease in Developing Countries*.

[24] Taye M., Berhanu T., Berhanu Y., Tamiru F., Terefe D. (2013). Study on carcass contaminating *Escherichia coli* in aparently healthy slaughtered cattle in Haramaya university slaughter house with special emphasis on *Escherichia coli* O157:H7. *Journal of Veterinary Science and Technology*.

[25] Abdissa R., Haile W., Fite A. T. (2017). Prevalence of *Escherichia coli* O157:H7 in beef cattle at slaughter and beef carcasses at retail shops in Ethiopia. *BMC Infectious Diseases*.

[26] International Organization for Standardization (2003). *Microbiology of Food and Animal Feeding Stuffs -Carcass Sampling for Microbiological Analysis*.

[27] Clinical and Laboratory Standards Institute (2016). Performance for antimicrobial disk susceptibility testsing.

[28] Mohammed O., Shimelis D., Admasu P., Feyera T. (2014). Prevalence and antimicrobial susceptibility pattern of *E. Coli* isolates from raw meat samples obtained from abattoirs in Dire Dawa city, eastern Ethiopia. *International Journal of Microbiological Research*.

[29] Olatoye I. O., Amosun E. A., Ogundipe G. A. (2012). Multidrug resistant *Escherichia coli* O157 contamination of beef and chicken in municipal abattoirs of Southwest Nigeria. *Journal of Nature and Science*.

[30] Abebe M., Hailelule A., Abrha B. (2014). Antibiogram of *Escherichia coli* strains isolated from food of bovine origin in selected Woredas of Tigray, Ethiopia. *Journal of Bacteriology Research*.

[31] Hajian S., Rahimi E., Mommtaz H. (2011). A 3-year study of *Escherichia coli* O157:H7 in cattle, camel, sheep, goat, chicken and beef minced meat. *Food Engineering and Biotechnology*.

[32] Tahamtan Y. E., Pourbakhsh S. A., Shekarforoush S. S. (2006). PCR detection of *Escherchia Coli* O157:H7 directed from slaughtered cattle in Shiraz,Iran. *Journal of Archives Razi Institute*.

[33] Abong B. O. (2008). Prevalence of *Escherichia coli* O157:H7 in water, meat, meat products. *And Vegetables Sold in the Eastern Cape Province of South Africa and its Impact on the Diarrheic Conditions of HIV/AIDS Patients*.

[34] Zhang S., Zhu X., Wu Q., Zhang J., Xu X., Li H. (2015). Prevalence and characterization of *Escherichia coli* O157 and O157:H7 in retail fresh raw meat in south China. *Annals of Microbiology*.

[35] Mekonnen H., Habtamu T., Kelali A., Shewit K. (2013). Food safety knowledge and practices of abattoir and butchery shops and the microbial profile of meat in Mekelle City, Ethiopia. *Journal of Tropical Biomedicine*.

[36] Magwira C. A., Gashe B. A., Collison E. K. (2005). Prevalence and antibiotic resistance profiles of *Escherichia coli* O157:H7 in beef products from retail outlets in Gaborone, Botswana. *Journal of Food Protection*.

[37] EFSA (2013). The European union summary report on trends and sources of Zoonoses , trends and sources of Zoonoses , zoonotic agents and food-borne outbreaks in 2011. *EFSA Journal*.

[38] Dahiru M., Uraih N., Enabulele S., Shamsudeen U. (2008). Prevalence of Eschericia coli 0157:H7 in fresh and roasted beef in Kano City, Nigeria. *Bayero Journal of Pure and Applied Sciences*.

[39] Varela J. J., Cabrera-Diaz E., Cardona M. A (2007). Isolation and characterization of Shiga toxin-producing *E. coli* O157:H7 and non-O157 from beef carcasses at a slaughter plant in Mexico. *International Journal of Food Microbiology*.

[40] Abd-Elaleem R., Bakr W. M. K., Hazzah W. A., Nasreldin O. (2014). Assessment of the personal hygiene and the bacteriological quality of butchers’ hands in some abattoirs in Alexandria, Egypt. *Food Control*.

[41] Jianu C., Goleţ I. (2014). Knowledge of food safety and hygiene and personal hygiene practices among meat handlers operating in western Romania. *Food Control*.

[42] McIntyre L., Vallaster L., Wilcott L., Henderson S. B., Kosatsky T. (2013). Evaluation of food safety knowledge, attitudes and self-reported hand washing practices in FOODSAFE trained and untrained food handlers in British Columbia, Canada. *Food Control*.

[43] Nel S., Lues J. F. R., Buys E. M., Venter P. (2004). The personal and general hygiene practices in the deboning room of a high throughput red meat abattoir. *Food Control*.

[44] Mann I., Koulikovskii A., Matyas Z. (1984). Guidelines on small slaughterhouses and meat hygiene in developing countries.

[45] FAO (2004). Food and agriculture organization animal production and health.

[46] Elizabeth A. J. C., William A. G., Lian F. T., Samuel K., Barend M. C. B., Eric M. F. (2017). Working conditions and public health risk in slaughterhouses in western Kenya. *Journal of Public Health*.

[47] Bartz S., Ritter A. C., Tondo E. C. (2010). Evaluation of bacterial multiplication in cleaning cloths containing different quantities of organic matter. *The Journal of Infection in Developing Countries*.

[48] Jamaa P. A. (2005). Hand hygiene: simple and complex. *International Journal of Infectious Diseases*.

[49] Fawzi M., Gomaa N. F., Bakr W. M. (2009). Assessment of hand washing facilities, personal hygiene and the bacteriological quality of hand washes in some grocery and dairy shops in Alexandria, Egypt. *Journal of the Egyptian Public Health Association*.

[50] WHO (2009). *Food Hygiene Basic Texts*.

[51] Codex-Alimentarius-Commission (2005). Code of hygienic practice for meat.

[52] Nørrung B., Buncic S. (2008). Microbial safety of meat in the European Union. *Journal of Meat Science*.

[53] Gill C. O., McGinnis J. C., Badoni M. (1995). Assessment of the hygienic characteristics of a beef carcass dressing process. *Journal of Food Protection*.

[54] Nastasijevic I., Mitrovic R., Buncic S. (2008). Occurrence of *Escherichia coli* O157 on hides of slaughtered cattle. *Journal of Letters in Applied Microbiology*.

[55] Blagojevic B., Antic D., Ducic M., Buncic S. (2011). Ratio between carcass-and skin-microflora as an abattoir process hygiene indicator. *Food Control*.

[56] Ethiopia FNGoTFDRo (2010). Proclamation to provide for food, medicine and health care administration and control. *No. 661/2009*.

[57] Rahimi E., Nayebpour F. (2012). Antimicrobial resistance of *Escherichia coli* O157:H7 isolated from feaces of ruminant animals in Iran. *Journal of Cell and Animal Biology*.

[58] Singh R., Schroeder C. M., Meng J. (2005). Identification of antimicrobial resistance and class 1 integrons in Shiga toxin-producing *Escherichia coli* recovered from humans and food animals. *Journal of Antimicrobial Chemotherapy*.

[59] Salehi T. Z., Bonab S. F. (2006). Antibiotics susceptibility pattern of *Escherichia* coli strains isolated from chickens with coli septicemia in Tabriz Province, Iran. *International Journal of Poultry Science*.

[60] Guerra B., Junker E., Schroeter A., Malorny B., Lehmann S., Helmuth R. (2007). Phenotypic and genotypic characterization of antimicrobial resistance in German *Escherichia coli* isolates from cattle, swine and poultry. *Journal of Antimicrobial Agents Chemotheraphy*.

[61] Akond M. A., Alam S., Hasan S. M., Mubassara S., Uddin S. N., Shirin M. (2009). Antibiotic resistance of *Escherichia coli* isolated from poultry and poultry environment of Bangladesh. *American Journal of Environmental Science*.

